# Carbon footprint of a clinical biochemistry platform in a French university hospital: Identifying the main greenhouse gas emission sources

**DOI:** 10.1016/j.joclim.2026.100686

**Published:** 2026-05-23

**Authors:** Klinguer Cécile, de Viry Margaux, Rimlinger Florence, Leroy Chrystel, Borderie Didier, Chenevier-Gobeaux Camille

**Affiliations:** a Département Transformation écologique et Santé environnementale, Direction de la Stratégie et de la Transformation, APHP, Paris, France; b Service de Biochimie, Hôpital Cochin, APHP, Paris, France

**Keywords:** Carbon footprint, Clinical laboratory, Greenhouse gas, Decarbonization of medical activities, Hospital, Health system

## Abstract

**Background:**

The healthcare sector is a significant contributor to greenhouse gas (GHG) emissions, accounting for approximately 8% in France. Clinical biochemistry laboratories, crucial for patient diagnosis and therapeutic follow-up, are particularly resource-intensive and the laboratory consumables represent the second biggest emission source in Parisian public hospitals.

**Methods:**

We aimed to quantify the carbon footprint of the centralized analytical automated platform of the clinical biochemistry laboratory at Cochin Hospital in Paris, covering most of the incoming and outgoing physical flows based on actual operational data. Wherever possible, physical emission factors were used to assess the emissions associated with laboratory activities. For laboratory consumables, monetary emission factors were applied due to the lack of available data.

**Findings:**

The carbon footprint assessment revealed that the laboratory's COBAS® automated analytical lines emitted 2081 tCO₂e in 2023, equivalent to approximately 0.52 kgCO₂e per test. The study found that laboratory consumables accounted for nearly 80% of total emissions. A comparison with other studies showed similar orders of magnitude though it highlighted methodological differences affecting the results. The analysis by analytical module revealed significant variations in emissions, with the immunoassay module having the highest footprint per test (2.89 kgCO₂e/test), primarily driven by expensive laboratory consumables.

**Interpretation:**

Our study provides valuable insights into the GHG emissions of a centralized analytical automated platform and suggests that decarbonization efforts should focus on reducing consumable waste and promoting appropriate test ordering practices. Further research is needed to estimate emissions associated with specific tests and incorporate sample collection and transport emissions.

## 1 Introduction

The 2024 Lancet Countdown report stipulates that the health threats posed by climate change are greater than ever: among the 15 indicators used to track the health impacts of climate change, 10 reached record levels in their most recent year of assessment [1]. Climate-related events are placing increasing pressure on health systems. Despite this growing threat, only 0.5% of climate change projects that receive funding from international organizations are dedicated to protecting human health [2].

Yet the healthcare system itself contributes to global warming. In member countries of the Organization for Economic Cooperation and Development (OECD), in China, and in India, greenhouse gas (GHG) emissions from the healthcare sector account for an average of 5% of national emissions, a carbon footprint comparable to that of the food sector in those countries [3]. In France specifically, a study by The Shift Project, a think tank dedicated to the decarbonization of all economic sectors, estimates that the healthcare sector is responsible for approximately 8% of the country's total GHG emissions [4].

Within this sector, clinical biochemistry laboratories play a critical role in patient diagnosis and therapeutic follow-up. However, they are particularly resource-intensive structures, reportedly using up to 10 times more energy and 4 times more water than comparable commercial buildings [5]. This is largely due to the wide range of energy-demanding equipment they rely on (e.g., analyzers, centrifuges, fume hoods, cold rooms). These facilities also heavily depend on single-use devices and involve the use of complex chemical reagents.

To date, few studies have specifically addressed the environmental impact of clinical biochemistry laboratories. Some studies have investigated the hospital scale; within Assistance Publique-Hôpitaux de Paris (AP-HP) hospital network, laboratory consumables were identified as the second largest source of emissions among hospital activities, following pharmaceuticals [6]. Most existing research has focused on carbon footprint of pathology testing in an Australian laboratory [[7], [8], [9], [10], [11]]. A carbon footprint assessment was conducted in a pathology laboratory in Lille (France) but did not account for all laboratory consumables [12]. Though some initiatives dedicated to decarbonizing clinical laboratory emerge, such as the ‘Let’s decarbonize clinical chemistry’ (‘*Décarbonons la biologie médicale*’) program of the French Society for Clinical Biology, the environmental impact of laboratories and their potential decarbonization levers are not as widely identified.

This study aims to quantify the carbon footprint of the centralized analytical automated platform of the clinical biochemistry laboratory at Cochin Hospital, which is part of the AP-HP network in Paris. The approach covers most incoming and outgoing physical flows, based on actual operational data, and uses a mix of physical emission factors (when available) and monetary ratios to estimate the associated GHG emissions. The analysis also provides an estimate of the average carbon footprint of multiple tests performed on the COBAS® modules composing the analytical automated platform of the clinical biochemistry laboratory.

## 2 Materials and methods

### 2.1 Study design

This study aimed to quantify the complete carbon footprint of the clinical biochemistry laboratory that conducts biology examinations on COBAS® modules at the Cochin Hospital laboratory. The objective was to quantify the average GHG emissions associated with multiple tests performed on the analytical chain, and to assess the variation in emissions according to the type of COBAS® module involved (c502, c701, e801, and ISE). This work does not intend to provide a specific GHG emission value for each individual analyte or test parameter, but rather to offer a global and comparative view of emissions across the automated platform.

### 2.2 Methodological framework

The methodology followed the official guidelines published in July 2022 by the French Ministry for Ecological Transition and ADEME (the French Agency for Ecological Transition) [13,14]. Emissions were calculated by multiplying activity data - such as quantities of consumables, energy use, and equipment operation - with corresponding emission factors expressed in kilograms of CO₂ equivalent per unit. Each emission estimate (E in the formula below) was associated with its corresponding uncertainty, calculated following Intergovernmental Panel on Climate Change (IPCC) guidelines and taking into account both data collection uncertainty (U_data_) and emission factor uncertainty (U_EF_) [15]:

$$U_{total}=\frac{\sqrt{\sum_{n}\left(U_{data,n}^{2}+U_{EF,n}^{2}\right)\times E_{n}^{2}}}{\sum_{n}E_{n}}$$

### 2.3 Scope of the study

**Fig. 1:**
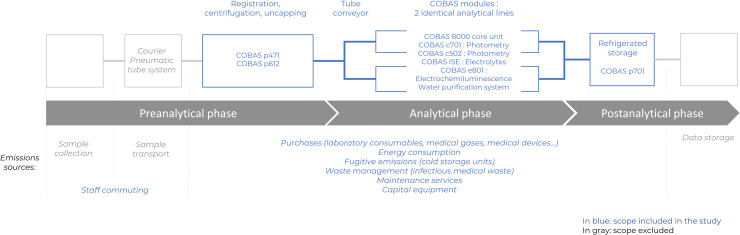
Scope of the study. The figure describes the main steps of the studied process – pre-analytical, analytical and post-analytical phases, as well as the emissions sources included in the study. Those include the laboratory purchases, energy consumption, fugitive emissions from the cold storage units, staff commuting, capital equipment and maintenance services and infectious medical waste management. Each box represents a step of the process; lines represent the conveyors between each phase. In blue, scope included in the study. In gray, scope excluded.

The study focused on the Cochin Hospital's clinical biochemistry laboratory and covered the operational process of the COBAS® automated platform during the year 2023, from the pre-analytical to the post-analytical phase (Fig. 1).

− The scope of the pre-analytical assessment was limited to the in-laboratory phase, including the initial steps of sample processing - registration, centrifugation, and uncapping - as well as the transport of tubes between modules using automated conveyors. Transport of samples from the services to the laboratory (whether by courier or pneumatic tube systems) and sample collection occurring outside the laboratory were excluded due to data limitations;
− the analytical processes performed by various conveyed COBAS® modules (2 c701, 2 c502, 3 e801, 2 ISE) were integrated, including the auxiliary systems such as the water purification unit of the analytical lines;
− the post-analytical studied perimeter was limited to the refrigerated sample storage. Due to data limitation, data storage, result interpretation and reporting, and sample disposal (except for infectious waste) were excluded.

### 2.4 Data collection

Emission sources were identified and categorized according to their nature and relevance to the functioning of the COBAS® analytical platform. The analysis included emissions directly linked to laboratory infrastructure, as well as those associated with energy use and operational logistics.

The data were collected in close collaboration with the Cochin Hospital laboratory staff and the AP-HP central services. Each category of emissions was addressed individually, using institution-specific sources when available, and completed with standard methodological assumptions where necessary.

#### 2.4.1 Laboratory consumables

A significant portion of the emissions were linked to the purchase and use of laboratory consumables. Procurement data were used to identify items purchased by the lab, and Cochin hospital lab staff pointed out the items used specifically in COBAS® operations. These items were then matched with the emission factors used in the AP-HP institutional carbon footprint, which are primarily based on the national Base Empreinte® database (and in the case of laboratory consumables, are mainly monetary ratios) and complemented, when possible, by international datasets [16]. Emissions were allocated to each COBAS® module according to the tests processed on that module. Items provided free of charge—such as certain cleaning supplies—were excluded from the total; however, approximate carbon footprint based on their estimated market price confirmed that their contribution would have been negligible.

#### 2.4.2 Medical gases, medical devices, transportation and other purchases

The same methodology was applied to medical gases, medical devices, transportation services, and other purchases of the clinical biochemistry laboratory necessary to operate the COBAS® platform. Data were retrieved from the AP-HP purchasing system, and each product or service was matched with its corresponding emission factor as used in the AP-HP institutional carbon footprint. For medical devices, physical emission factors from the AP-HP institutional carbon footprint were used where available [6]; otherwise, a monetary-based approach was used, with emissions calculated by applying standard emission factors per euro spent [4].

#### 2.4.3 Energy consumption

Electricity consumption was estimated by calculating the power usage of each COBAS® module, based on technical specifications of the equipment. All devices were assumed to operate 22 h per day, 7 days per week throughout the year, to reflect real-life continuous operation, including during inactive periods. Heating emissions from the CPCU (*Compagnie Parisienne de Chauffage Urbain*, the Paris district heating network) were estimated using a surface-area-based ratio derived from the AP-HP carbon footprint for the Cochin site.

#### 2.4.4 Fugitive emissions

Refrigerant emissions were taken into account for the two cold storage units associated with the COBAS® chain. The refrigerant fluid charge and type were obtained from the hospital’s maintenance and safety service. Emissions were then calculated based on standard leakage rates and the global warming potential of the refrigerants used.

#### 2.4.5 Staff commuting

Emissions from staff commuting were estimated using the number of full-time equivalent (FTE) staff in the laboratory, under the assumption that all personnel contributed to COBAS®-related activities. An average emission factor per FTE was applied, based on the 2023 Cochin site emissions data.

#### 2.4.6 Maintenance services

All service-related purchases identified in the AP-HP procurement system were included, following the same classification system used in the institutional carbon footprint. Preventive maintenance for the COBAS® modules was estimated at 8 h per month for the 13 modules, evenly distributed across modules, with emissions calculated using standard service-related emission factors from the national Base Empreinte® database.

#### 2.4.7 Capital equipment

The COBAS® modules were considered as capital goods, and emissions were estimated based on approximate acquisition values, as the equipment is provided to Cochin’s clinical biochemistry laboratory under leasing arrangements. Estimates assumed installation in 2013 for the c502, c701 and ISE modules, in 2017 for the e801 and a 10-year depreciation period, following the national methodology for capital equipment.

#### 2.4.8 Waste management

Only emissions related to the management of infectious medical waste were included. Daily volumes of infectious waste generated by COBAS® -related activities were estimated based on staff input and typical waste production patterns. Emissions from other waste types were not considered, as they are negligible in the AP-HP institutional carbon footprint (<0.5% of the global carbon footprint) and thus, have been considered to be negligible as well in this study.

#### 2.4.9 Digital services

Digital emissions were calculated based on IT-related purchases identified in the procurement records, in accordance with the categories used in the institutional methodology. Emissions related to data storage and digital result archiving were excluded due to the lack of quantifiable data.

### 2.5 Data analysis

Consumables were attributed to specific tests types based on their actual use, except for those used across all COBAS® modules, which emissions were evenly distributed. Emissions from energy use, transport, maintenance, waste, and equipment were distributed proportionally to the number of tests conducted. This enabled both a global assessment of the COBAS® platform and a more granular examination of representative biochemistry tests.

When physical emission factors were not available to estimate the emissions associated with some categories in the study’s perimeter (for example the laboratory consumables), monetary ratios had to be used. Though they are associated with a higher uncertainty than physical emission factors, they make it possible to include all emissions in the study’s perimeter [16].

### 2.6 Statistical analysis

Continuous variables are presented as median [interquartile range, IQR], categorical variables as numbers and percentages. Continuous variables were compared with the Kruskal-Wallis test, and categorical variables using the Pearson chi-square test. Correlations among continuous variables were assessed with the use of the Spearman rank-correlation coefficient. All tests were 2-tailed, and a p value of <0.05 was considered significant. Statistical analysis was performed using MedCalc 19.3.1 for Windows (MedCalc Software. Mariakerke, Belgium).

## 3 Results

### 3.1 Total emissions according to the emission source

Total 2023 emissions were 2081 tCO₂e (uncertainty 19%). The number of tests performed in the COBAS® 8000 in 2023 was 3986,424, thus the emission per test was 0.52 kgCO₂e/test.

**Fig. 2:**
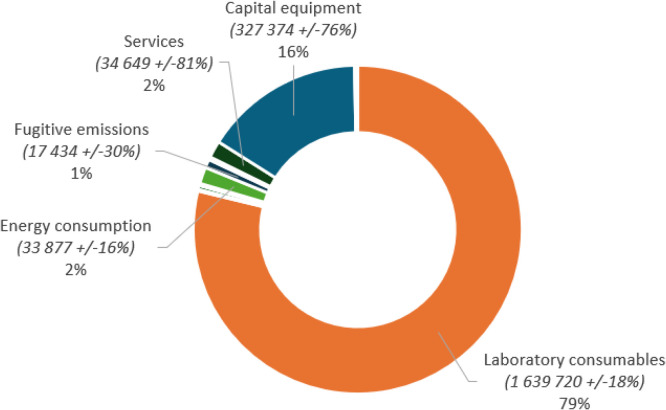
Distribution of the GHG 2023 emissions of the COBAS® analytical lines per emission source (in kgCO_2_e ± uncertainty %). The figure presents the distribution of the GHG emissions per emission sources while specifying the absolute value of the emissions (in kgCO_2_e ± the uncertainty associated to each source) and the relative share of each emission source compared to the total.

Fig. 2 shows the distribution of emissions according to each source. The laboratory consumables represent almost 80% of the total emissions, and the second main source of emission was capital equipment (16%). Of note, the annualized emission for energy was 33 tCO₂e for our platform.

### 3.2 Total emission according to analytical step

An analysis was conducted to assess the emissions per analytical module.

#### 3.2.1 List of studied tests, modules and corresponding annual test volume

The list of studied tests, modules and corresponding annual test volume is presented in the Supplementary Table 1. The clinical biochemistry COBAS® platform performed 119 different tests across 4 different analytical modules. Annual test volumes ranged from 36 to 1,308,168.

#### 3.2.2 Correlation of total emissions with test volume

The total emission was moderately but significantly correlated to the annual test volume. We found a correlation coefficient r equal to 0.333 (*p* < 0.001); tests performed in high quantity annually are those having highest emissions. The relationship between tests volume and total emission is apparent, but it is not very strong. The variables are linked, but other factors may have a significant influence on the results.

#### 3.2.3 Total emissions according to type of analytical module

We further studied the total emissions according to the type of analytical module used to perform the tests (Table 1). The module that has the greater total emission is the E801 (immunoassay module), and this represents 50.8% of the total emission; the module that has the lowest emission is the ISE. The immunoassay module remains the first productor of kgCO₂e when considering emissions per test.

**Table 1:** Carbon dioxide equivalent emissions (tCO_2_e) associated with each analytical COBAS module.

Carbon dioxide equivalent emissions	COBAS c502	COBAS c701	COBAS E801	COBAS ISE
**TOTAL 2023 (tCO_2_e)**	369	588	1057	67
**TOTAL / test (kgCO_2_e/test)**	0.685	0.202	2.890	0.399

**Fig. 3:**
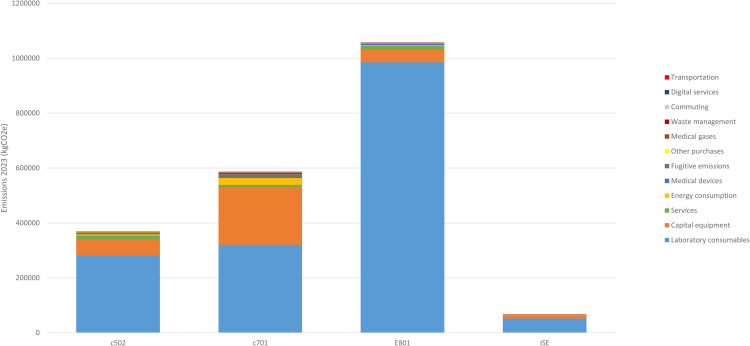

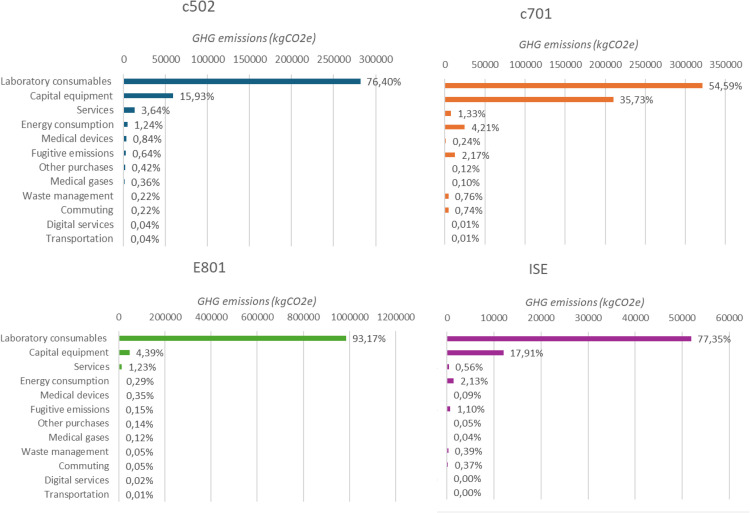
a: Cumulative distribution of 2023 GHG emissions per COBAS® module. Distribution of the GHG emissions per emission source for each COBAS® module on a cumulative histogram that allows comparison and shows the absolute difference between the total emissions for each module. b: Detailed distribution of GHG emissions per sources for each COBAS® module. Histogram of the GHG emissions (x-axis) per emission source (y-axis) for each COBAS® module on separated graphs so that the share of each emission source is more visible.

We also analyzed the contribution of emissions sources (medical gases, medical devices, transport, other purchases, energy consumption, fugitive emissions, staff commuting, services except COBAS® maintenance, waste management and digital services) for each analytical module (Fig. 3). Laboratory consumables remain the primary source of emissions across all types of analytical modules. For the E801 module specifically, the laboratory consumables accounted for 984,950 kgCO₂e out of a total of 1,057,000 kgCO₂e (i.e., 93%).

### 3.3 Total emissions according to the tests

We finally studied the total emissions per test and presented the ten most CO₂e-intensive tests of the analytical platform (Table 2). Seven tests were performed on the E801 modules. The higher annual volume tests were for Electrolytes and C-reactive protein. The most CO₂e-intensive test was NT-proBrain Natriuretic peptide.

**Table 2:** Top ten most CO₂e-intensive tests.

Test	Total Emissions (kgCO_2_e)	Annual number of tests performed in 2023	Analitycal Module (analyzer) performing the test
NT-pro Brain Natriuretic peptide	691,970	17,784	E801
Procalcitonin	305,674	4188	E801
C-Reactive protein	218,075	112,476	c701
Ferritin	175,433	37,296	E801
Electrolytes (Na, K, Cl)	129,510	168,324	ISE
High-sensitivity cardiac troponin	127,022	21,192	E801
beta hCG	117,613	1356	E801
folates	94,454	12,588	E801
Biliary acids	83,855	480	c502
CA19–9	79,105	1392	E801

## 4 . Discussion

The study conducted at the clinical biochemistry laboratory of Cochin Hospital found that the carbon footprint of its two COBAS® automated analytical lines amounted to 2081 tCO₂e in 2023. During the same period, the laboratory processed 3986,424 tests, with an estimated average emission of approximately 0.52 kgCO₂e per test on these systems. We also identified the main sources of emissions in the operational process of the clinical laboratory. These results are highly dependent on the characteristics of the laboratory, particularly its activity level, which influences how fixed emissions (such as those from capital equipment, quality control reagents, and shared resources) are allocated per test, its geographical location, and its operating procedures.

While our results are novel regarding analyzer platform and geographic location, our study can be compared to others reported in the literature. Most of the literature explored anatomopathological laboratories, which have different organization in test processing form our practice. First, a study by McAlister et al. estimated the carbon footprint of six pathology tests in Australia, found results ranging from 0.074 kgCO₂e/test to 0.538 kgCO₂e/test [8]. These observed ranges of emissions highlight important methodological differences that affect the results (though the order of magnitude in this study aligns with our own). Their analysis was based on a consequential life cycle assessment approach, meaning that only the marginal emissions attributable to each test were considered. As such, emissions related to the general functioning of the laboratory, such as background energy consumption or shared reagents and use of consumables, were excluded. This contrasts with our attributional approach, which includes all operational emissions. Secondly, their results indicate that the majority of CO₂e emissions (up to 95% for coagulation tests) were associated with sample collection. In our study, sampling and transport were excluded from the system boundaries, which may partially explain differences in the distribution of emissions sources. Finally, in our analysis, the use of monetary emission factors to estimate the impact of laboratory consumables may have led to an overestimation of their carbon footprint, compared to McAlister et al., who estimated reagent emissions using molecular-level emission factors adjusted by a multiplier (×25) to account for chemical purity [8]. Our results are also of a similar order of magnitude to those of Trécourt et al.*;* although they focused on a different sample type (biopsies processed in anatomic pathology), their estimated total carbon footprint of 0.363 kgCO₂e - 1.481 kgCO₂e per test is consistent with our results [9]. However, the breakdown of emission sources differs: staff travel represented a larger share in their results, while our study, involving a much higher volume of tests per staff member, shows a greater contribution from reagents and consumables. In the same field of laboratory specialty, another American study conducted by Gordon et al. quantified the GHGs associated with processing a gastrointestinal biopsy [17]. These authors found emissions from biopsy processing between 0.290 and 0.790 kgCO₂e, and identified that production of supplies and reagents were the largest contributors to emissions, similar to our findings. A 2023 study by Spoyalo et al. estimated the emissions associated with processing a common panel of blood tests at 332 gCO₂e; adding a panel of liver tests resulted in an additional emission at 462 gCO₂e [18]. This observation is very close to our own findings [18]. In this study, the test processing was closest to our practice. However, the distribution of these emissions differs significantly; in their study, the vast majority of emissions related to chemistry testing are linked to energy consumption. The variation in carbon intensity between the French and Canadian electricity grids might explain this discrepancy.

Other studies have explored the energy consumption of clinical laboratories. Bandari et al. estimated the solid waste production and energy-related CO_2_ emissions for five major high-throughput chemistry analyzer systems, including a Roche platform similar to the one in our study [19]. Although their objectives and methodology differed from ours, they reported annualized CO₂e for energy (27.6 tCO₂e for the Roche platform) that were comparable to our findings (33 tCO₂e). Notably, they observed emission variations across different platforms. However, a definitive comparison remains difficult, as the operating timespan and raw electricity consumption of the platform in the Bandari et al. study were not specified. Additionally, waste production cannot be compared between the two studies, as our measurement was limited to infectious waste. Another study from Ni et al. assessed the daily electricity consumption of a laboratory's infrastructure, including HVAC systems, lighting, computers, and the production of plastic and paper waste [20]. However, their findings are not easily comparable to ours as we evaluated the impact of laboratory infrastructure energy consumption as a proportional share of the hospital’s total energy-related emissions. In the study by Ni *et al*., it is not possible to isolate the impact of the analyzer from that of other infrastructure systems. Furthermore, we excluded plastic and paper waste, as their GHG emissions were considered negligible in comparison to those of infectious waste. Our study therefore provides valuable insight into the GHG emissions associated with clinical tests in France, encompassing all primary emission sources.

The carbon footprint assessment by analytical module revealed notable differences in emissions between modules. In 2023, total GHG emissions were estimated at 369 tCO₂e for the c502 module, 588 tCO₂e for c701, 1,057 tCO₂e for e801, and 67 tCO₂e for ISE. When normalized per test, emissions were highest for the e801 module (2.89 kgCO₂e/test), significantly exceeding those of c502 (0.685 kgCO₂e/test), ISE (0.399 kgCO₂e/test), and c701 (0.202 kgCO₂e/test). This elevated footprint for e801, which uses electrochemiluminescence technology, is primarily driven by laboratory consumables, especially expensive due to the complexity of the analytical method, rather than capital equipment. As the laboratory consumables emissions are estimated with monetary ratios, a bias possibly exists that artificially increases the e801 emissions as the related consumables are more expensive.

We believe that our study shed light on two main issues: first, it is essential for laboratories to identify their primary sources of GHG emissions, as this is a prerequisite for developing a relevant and targeted decarbonization strategy. Previous studies have rarely pinpointed these major sources, making it difficult for environmentally conscious laboratory staff to identify effective actions to reduce their professional environmental impact. Second, while laboratory consumables contribute a substantial portion of a hospital’s carbon footprint, few studies have estimated the carbon footprint of biological tests. The estimates provided in our study could help clinicians consider the environmental impact of their test ordering strategies and the carbon footprint of patient care pathways, as demonstrated recently in an intensive care unit [21]. In practice, our findings could assist laboratories in better managing their emissions and consumption by suggesting specific decarbonization actions that align with established laboratory stewardship principles, such as:

− Reducing the purchase of laboratory consumables through improved inventory management (and thus waste reduction),
− Limiting the number of tests performed for quality control and calibration,
− Minimizing redundant or unnecessary tests.

Targets can be set for monitoring purposes, and these data also can support inter-laboratory comparisons of environmental performance.

The main limitation of this study lies in the use of monetary emission factors. These were applied in the calculation of the carbon footprint associated with laboratory consumables, some medical devices, capital goods, office supplies, and outsourced services purchased by the laboratory. This approach was necessary to address the lack of available data, as physical emission factors for these items are currently not provided by suppliers, public databases, nor via the internal emission factor database developed at AP-HP. While monetary emission factors are less precise (80% uncertainty) than physical ones (10–50% uncertainty range for the physical emission factors used in our study, reflecting the usual uncertainties of emission factors in the Base Empreinte® database) [16], they nonetheless enable the inclusion of the full scope of the laboratory’s purchases, including products for which no specific physical emission factor is known. Furthermore, while monetary emission factors are not recommended for high-precision product assessments [22], they provide valuable insights into the order of magnitude for extensive inventories, such as the one for laboratory consumables analyzed in this study. It will be essential to refine these estimates as more granular and specific physical emission factors for laboratory consumables become available, so that the global uncertainty obtained (19%) can be reduced.

Additionally, one of the assumptions in the module-based analysis involves allocating CO₂e emissions proportionally to the number of tests performed on each analyzer. This approximation can be challenged, as such proportional allocation does not accurately reflect the consumption of shared resources across modules (e.g., cleaning agents or gloves, which are used throughout the laboratory but not necessarily depending on the number of tests per module).

A direction for further research would be to estimate the emissions associated with a specific test. This more targeted approach would reduce some uncertainties, such as the proportional allocation of emissions based on test volumes. It could also incorporate emissions associated with sample collection and transport that were excluded from the scope of this study. These studies are currently ongoing for some chosen tests at AP-HP.

In conclusion, our study provides valuable insights into the GHG emissions of a French clinical biochemistry laboratory and suggests that decarbonization efforts should focus on reducing consumable waste and promoting appropriate test ordering practices. Further research is needed to estimate emissions associated with specific tests and incorporate sample collection and transport emissions.

## Funding

None.

## CRediT authorship contribution statement

**Klinguer Cécile:** Writing – review & editing, Writing – original draft, Supervision, Methodology, Formal analysis, Data curation, Conceptualization. **de Viry Margaux:** Writing – original draft, Methodology, Formal analysis, Conceptualization. **Rimlinger Florence:** Writing – original draft, Resources, Data curation. **Leroy Chrystel:** Writing – original draft, Resources, Methodology, Data curation. **Borderie Didier:** Writing – original draft, Supervision, Resources, Project administration. **Chenevier-Gobeaux Camille:** Writing – review & editing, Writing – original draft, Visualization, Validation, Supervision, Resources, Project administration, Formal analysis, Data curation.

## Declaration of competing interest

The authors declare the following financial interests/personal relationships which may be considered as potential competing interests:

Camille Chenevier-Gobeaux reports a relationship with Roche Diagnostics that includes: speaking and lecture fees and travel reimbursement. If there are other authors, they declare that they have no known competing financial interests or personal relationships that could have appeared to influence the work reported in this paper.

## Appendix Supplementary materials

Image, application 1

## Footnotes

Supplementary material associated with this article can be found, in the online version, at doi:10.1016/j.joclim.2026.100686.
