# Supplementary material for: Carbon footprint of a clinical biochemistry platform in a French university hospital: Identifying the main greenhouse gas emission sources
Source: J Clim Chang Health. 2026 May 23;29:100686. doi: 10.1016/j.joclim.2026.100686 (PMC13224059; doi:10.1016/j.joclim.2026.100686)
Supplement: Supplementary file 1 [file mmc1.docx]

**Supplementary Table I:** Tests, analytical module and annual test volume

| **Test**  (alphabetical order) | **Analitycal Module (analyzer) performing the test** | **Annual number of tests performed in 2023** |
| --- | --- | --- |
| Acetaminophen | c502 | 588 |
| Alpha-1 macroglobulin | c502 | 324 |
| Alpha-foeto protein | E801 | 3,108 |
| ALAT | c701 | 103,332 |
| Albumin | c701 | 135,552 |
| Alkalin phosphatase | c502 | 254,760 |
| Aldolase | c502 | 936 |
| Amikacin | c502 | 216 |
| Ammonia | c502 | 252 |
| Amylase | c502 | 936 |
| Angiotensin-converting enzyme | c502 | 3,720 |
| Anti-CCP | E801 | 4,224 |
| Anti-Mullerian hormone | E801 | 3,828 |
| anti-Thyroglobulin | E801 | 4,092 |
| anti-Thyroperoxydase | E801 | 4,548 |
| Anti-toxoplasmosis Ig G | E801 | 20,124 |
| Anti-toxoplasmosis Ig M | E801 | 20,112 |
| Apolipoprotein A | c502 | 816 |
| Apolipoprotein B | c502 | 732 |
| ASAT | c701 | 103,464 |
| Benzodiazepine | c502 | 420 |
| Beta hCG | E801 | 1,356 |
| Biliary acids | c502 | 480 |
| Buprenorphine | c502 | 240 |
| Crosslaps | E801 | 3,204 |
| Conjugated bilirubin | c701 | 13,080 |
| CA125 | E801 | 828 |
| CA15-3 | E801 | 432 |
| CA19-9 | E801 | 1,392 |
| CA72-4 | E801 | 108 |
| Caffeine | c502 | 276 |
| Calcium | c701 | 79,056 |
| Calprotectin | E801 | 120 |
| Carbamazepine | c502 | 108 |
| Carcino-embryonary antigen | E801 | 1,704 |
| Carnitine | c502 | 144 |
| Cholesterol | c502 | 31,944 |
| Ciclosporin | E801 | 168 |
| Citrate | c502 | 144 |
| Creatine kinase | c701 | 29,700 |
| CO_2_ | c701 | 149,268 |
| Cocaine | c502 | 36 |
| Cortisol | E801 | 5,628 |
| C- reactive protein | c701 | 112,476 |
| Creatinine | c701 | 209,064 |
| Digoxin | c502 | 60 |
| Electrolytes (Na, K, Cl) | ISE | 168,324 |
| Estradiol | E801 | 22,428 |
| Ethanol | c502 | 1,308 |
| Ferritin | E801 | 37,296 |
| Folates | E801 | 12,588 |
| Erythrocytary folates | E801 | 5,700 |
| Free T3 | E801 | 11,784 |
| Free T4 | E801 | 18,840 |
| Free-beta hCG | E801 | 6,456 |
| Free PSA | E801 | 2,724 |
| Fructosamine | c502 | 3,444 |
| Fructose | c502 | 120 |
| FSH | E801 | 5,628 |
| Gamma-glutamyltranspeptidase | c701 | 103,620 |
| Gentamicin | c502 | 72 |
| Glucose | c502 | 48,840 |
| Haptoglobin | c701 | 8,424 |
| HDL-cholesterol | c502 | 1,068 |
| High-sensitive cardiac troponin | E801 | 21,192 |
| human epididymis protein (HE)4 | E801 | 132 |
| Immunoglobulin A | c502 | 15,900 |
| Immunoglobulin G | c502 | 15,900 |
| Immunoglobulin M | c502 | 15,852 |
| Interleukin-6 | E801 | 768 |
| Iron | c502 | 19,752 |
| Lactates | c701 | 1,164 |
| Lactate deshydrogenase | c701 | 30,996 |
| LH | E801 | 20,364 |
| Lipase | c701 | 15,492 |
| Lithium | c502 | 924 |
| Magnesium | c701 | 28,032 |
| Met-amphetamine | c502 | 252 |
| Methotrexate | c502 | 1,584 |
| NT-pro Brain Natriuretic peptide | E801 | 17,784 |
| Osteocalcin | E801 | 2,040 |
| Opioids | c502 | 384 |
| Parathormon | E801 | 5,940 |
| Phenobarbital | c502 | 72 |
| Phenytoin | c502 | 120 |
| Phosphates | c502 | 64,644 |
| Placental growth factor (PlGF) | E801 | 1,632 |
| Pregnancy-associated plasma protein A (PAPP-A) | E801 | 1,344 |
| Procalcitonin | E801 | 4,188 |
| Progesterone | E801 | 19,608 |
| Prolactin | E801 | 5,736 |
| Rhumatoid factor | c701 | 5,280 |
| S100 protein | E801 | 288 |
| Salicilates | c502 | 72 |
| Sensitive methotrexate | c502 | 528 |
| serum cytokeratin 19 fragment (CYFRA21) | E801 | 48 |
| Serum/plasma indices (lipemia, hemolysis and jaundice) | c701 | 1,308,168 |
| Serum/plasma proteins | c701 | 196,608 |
| Sex hormon binding protein (SHBG) | E801 | 4,308 |
| Soluble fms-like tyrosine kinase 1 (SFLT-1) | E801 | 1,332 |
| Squamous Cell Carcinoma antigen (SCC) | E801 | 48 |
| Tacrolimus | E801 | 1,740 |
| Teicoplanin | c502 | 48 |
| Tetrahydrocannabinol (THC) | c502 | 384 |
| Thyroglobuline | E801 | 1,320 |
| Total bilirubin | c701 | 101,676 |
| Total mycophenolic acid | c502 | 516 |
| Total PSA | E801 | 3,984 |
| Tobramycin | c502 | 972 |
| Tricyclic acid | c502 | 1,020 |
| Transferrin | c701 | 19,812 |
| Triglycerids | c502 | 31,344 |
| TSH | E801 | 38,100 |
| Urea | c701 | 123,372 |
| Ureic acid | c502 | 15,192 |
| Urinary proteins | c701 | 36,192 |
| Valproate | c502 | 396 |
| Vancomycin | c502 | 540 |
| vitamin B12 | E801 | 15,432 |
| Zinc | c502 | 144 |
